# Sulfur microenvironments as hotspots for biogenic pyrite formation

**DOI:** 10.1038/s41598-025-05178-8

**Published:** 2025-06-20

**Authors:** Fatih Sekerci, Stefan Fischer, Prachi Joshi, Stefan Peiffer, Andreas Kappler, Muammar Mansor

**Affiliations:** 1 Geomicrobiology, Department of Geosciences, University of Tuebingen, Tuebingen, Germany; 2 Tuebingen Structural Microscopy Core Facility, University of Tuebingen, Tuebingen, Germany; 3 Hydrology, Bayreuth Center for Ecology and Environmental Science, University of Bayreuth, Bayreuth, Germany; 4 Cluster of Excellence: EXC 2124: Controlling Microbes to Fight Infection, Tuebingen, Germany

**Keywords:** Biogenic pyrite formation, Pyrite spherules, Microenvironment, Polysulfide, Sulfidic conditions, *Geobacter sulfurreducens*, Element cycles, Biogeochemistry

## Abstract

Pyrite (FeS_2_) is the end-product of bacterial sulfur cycling in reduced sedimentary environments. Sulfate-reducing bacteria (SRB) are mostly considered for biogenic pyrite formation due to their significant contribution to sulfide production at ambient temperatures. However, most experiments using SRB for biogenic pyrite formation resulted in the formation of only metastable iron sulfide minerals such as mackinawite (FeS). In this study, we investigated the roles of elemental sulfur (S^0^) and microbial sulfur reduction for pyrite formation. To this end, we cultivated the Fe(III)- and sulfur-reducing bacterium *Geobacter sulfurreducens* in the presence of the Fe(III) (oxyhydr)oxide mineral ferrihydrite and elemental sulfur at two different Fe/S ratios (4:1 and 1:4). While only mackinawite formed in Fe/S: 4:1 experiments, sulfidic conditions in the Fe/S: 1:4 experiments favored greigite (Fe_3_S_4_) and pyrite formation via the polysulfide pathway. Morphological observations demonstrated that spherulitic pyrite particles formed at the surface of elemental sulfur, with clusters forming that preserved the original morphology of sulfur particles. Our results showed that sulfur-reducing bacteria could replace the role of SRB as a sulfide source and further showed that sulfur particles are likely hotspots for biogenic pyrite formation by creating polysulfide-rich microenvironments and by acting as templates for spatially heterogeneous pyrite precipitation in nature.

**Supplementary Information:**

The online version contains supplementary material available at 10.1038/s41598-025-05178-8.

## Introduction

Pyrite (FeS_2_) is the most abundant iron sulfide mineral on Earth’s surface, and its burial represents the major iron and sulfur sink in reduced sediments^1^. Pyrite is thermodynamically stable, and its stability contributes to its preservation through geological timescales. The formation and dissolution of pyrite are linked to global biogeochemical cycles of iron, sulfur, carbon, nitrogen, and trace metals in sedimentary environments^2–6^. Thus, sedimentary pyrite can be used to trace the co-evolution of Earth’s biogeochemical cycles and life^6–9^. Sulfur isotopic signatures, pyrite morphology, and organic matter trapped in pyrite particles may indicate the biogenicity of the mineral in the environment^8,10–12^. However, successful attempts to synthesize biogenic pyrite at ambient temperatures are limited^13–15^. Many experiments with microbial cultures or microcosms containing sulfur-metabolizing bacteria (especially sulfate-reducing bacteria) resulted in the formation of mackinawite (FeS) and/or greigite (Fe_3_S_4_), without further transformation to pyrite^16,17^.

Pyrite can form via the sulfidation of Fe(III) (oxyhydr)oxide minerals and/or via the transformation of precursor iron sulfide minerals through a combination of abiotic and biotic processes^18,19^. There are three main pathways for pyrite formation. At high Fe(III)/S(–II) ratios, pyrite can form on the surface of Fe(III) (oxyhydr)oxides via the “ferric-hydroxide-surface (FHS) pathway” with a lack of detectable intermediates in the aqueous phases^20,21^. Under sulfidic conditions, pyrite can form through the oxidation of iron monosulfides by H_2_S via the “H_2_S pathway” (Reaction 1)^22^, or by polysulfides (SS_n_^2−^) in the “polysulfide pathway” (Reaction 2)^23^.

1 $${\text{FeS }} + {\text{ H}}_{{2}} {\text{S }} \to {\text{ FeS}}_{{2}} + {\text{ H}}_{{2}}$$

2 $${\text{FeS }} + {\text{ SS}}_{{\text{n}}}^{{{2} - }} \to {\text{ FeS}}_{{2}} + {\text{ S}}_{{{\text{n}} - {1}}}^{{{2} - }}$$

Polysulfide species form by the reaction between dissolved sulfide and elemental sulfur (S^0^) (Reaction 3)^24^. At ambient temperatures, sulfide is mostly produced by bacterial activity^3^, and S^0^ can be produced via the oxidation of sulfide by Fe(III) or Mn(IV) oxide minerals in anoxic environments (Reaction 4)^1,25^. Elemental sulfur is a chemically stable intermediate in sedimentary environments that can even accumulate in certain locations (10–14 µmol/cm^3^ sediment)^26^. Microbial activities can actively produce and dissolve S^0^ via dissimilatory sulfur oxidation, reduction and disproportionation^27–29^, resulting in a dynamic pool of S^0^ and intermediate sulfur species such as polysulfides. Therefore, the presence of S^0^ favors pyrite formation via the polysulfide pathway.

3 $${\text{HS}}^{ - } + {\text{ SS}}_{{{\text{n}} - {1}}}^{0} \to {\text{ SS}}_{{\text{n}}}^{{{2} - }} + {\text{ H}}^{ + }$$

4 $${\text{2FeOOH }} + {\text{ HS}}^{ - } + {\text{ H}}_{{2}} {\text{O }} \to {\text{ 2Fe}}^{{{2} + }} + {\text{ S}}^{0} + {\text{ 5OH}}^{ - }$$

Previous studies have indicated that microbial reduction of Fe(III) mineral is thermodynamically more favorable than S^0^ reduction at neutral pH^30^, but the proximity of cells to either Fe(III) minerals or S^0^ may result in concurrent reduction of both^17^, demonstrating the significance of microenvironments. We hypothesize that elemental sulfur could act as micro-sized hotspots for biogenic pyrite formation. To test this, we incubated the Fe(III)- and S^0^-reducing bacterium *Geobacter sulfurreducens* with the Fe(III) (oxyhydr)oxide mineral ferrihydrite and elemental sulfur. Two different Fe/S ratios (4:1 and 1:4)—keeping the Fe concentration constant at 30 mM—were tested to determine the effect of elemental sulfur abundance on solution chemistry and the resulting mineralogy. The results showed that an increasing abundance of S^0^ has an accelerating and templating effect on biogenic pyrite formation.

## Results and discussion

### Fe/S: 4:1 experiments: mackinawite formation in ferruginous conditions

**Fig. 1:**
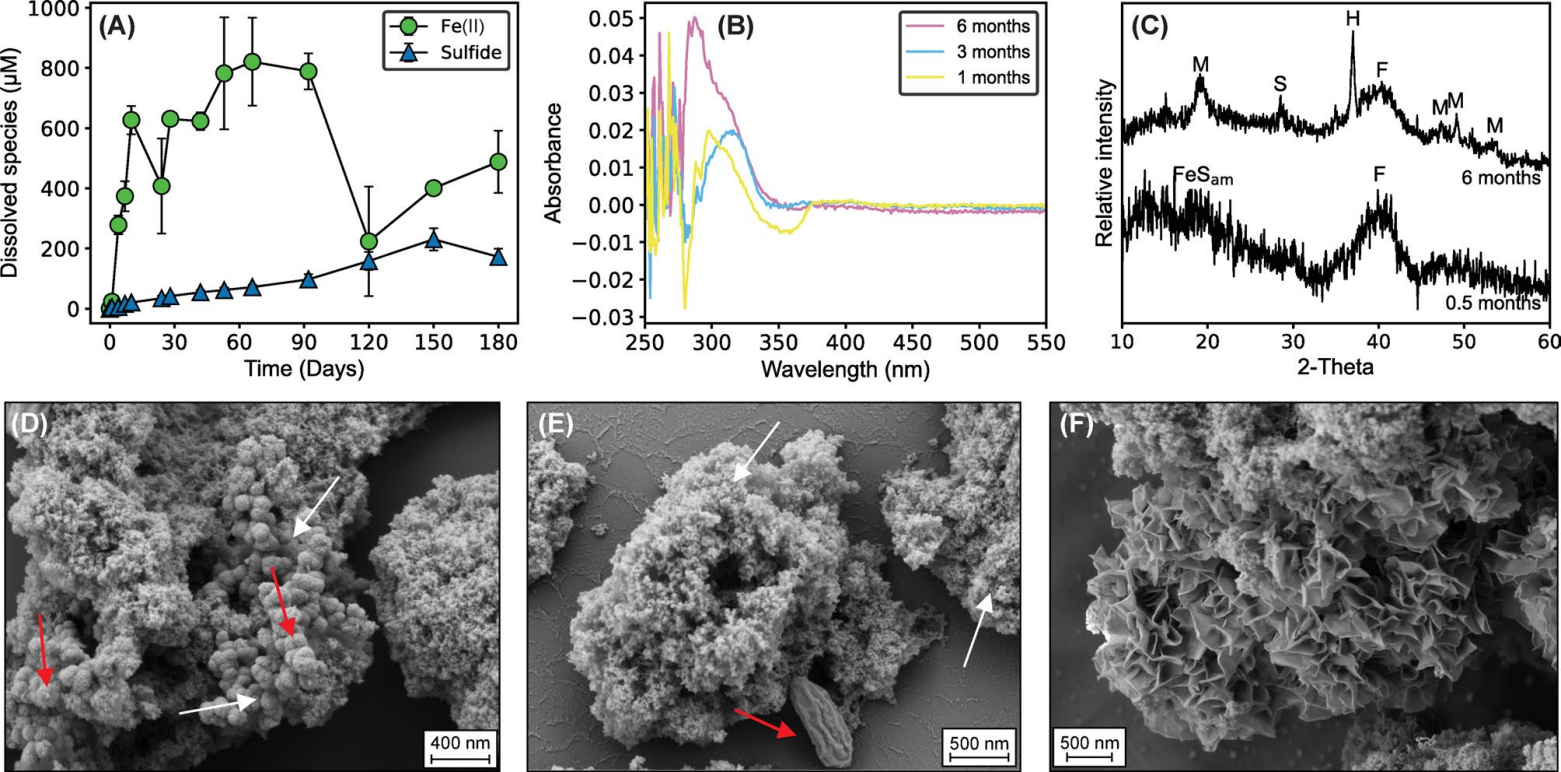
FeS_am_ formation and maturation to mackinawite in Fe/S: 4:1 experiments. (**A**) Dissolved Fe(II) and sulfide concentrations. (**B**) UV–Vis spectra for polysulfides. (**C**) Mineralogy of the particles by µXRD analysis. (D) SEM micrograph of anoxically-dried samples on day 15. Red arrows represent ferrihydrite particles still present in the system. White arrow points out FeS_am_ aggregates which are the initial products of the concurrent ferrihydrite and sulfur reduction. (**E**) SEM micrographs of chemically fixed samples on day 15. White arrows point out FeS_am_ aggregates, red arrow represents *G. sulfurreducens.* (**F**) FeS_am_ aggregates turned into a more crystalline mackinawite structure after 6 months. FeS_am_: Disordered mackinawite-like phases, M: mackinawite, F: ferrihydrite, S: sulfur, H: halite.

A color change from brown to black, indicative of ferrihydrite redox transformation, was observed in Fe/S: 4:1 experiments within the first 15 days (Supplementary Fig. 1). Dissolved Fe(II) accumulated rapidly within this period and reached a plateau of ~ 820 µM after the second month. Dissolved sulfide, originating from microbial sulfur reduction, also increased with the highest value of 230 µM at the fifth month, but remained less than Fe^2+^_(aq)_ during the 6-month experimental period (Fig. 1a). Dissolved polysulfide species were not detected in the UV–Vis spectra for the first 3 months. Small peaks at 290 nm for the 6th month sample may indicate the presence of a minor amount of polysulfides^10,31^, but the elevated background signal at 250–265 nm implies that the detection of polysulfides was inconclusive (Fig. 1b). Because mackinawite forms and precipitates rapidly in the presence of ferrous iron and sulfide due to its low solubility (log *K*_*sp*_ = − 5.70, Reaction 5)^1^, the Fe^2+^-rich solution in our Fe/S: 4:1 experiments favored the consumption of sulfide via mackinawite formation, rather than contributing to polysulfide formation.

5 $${\text{Fe}}^{{{2} + }} + {\text{ HS}}^{ - } \to {\text{ FeS }} + {\text{ H}}^{ + }$$

The formation of mackinawite was confirmed by µXRD analysis (Fig. 1c). SEM (Scanning electron microscopy) micrographs for the samples on day 15 suggested that ferrihydrite was still present in the system (Fig. 1d), and secondary sulfide minerals were identified by their morphology as small aggregates at the limit of SEM resolution (Fig. 1d–e). The broad signal at 18° 2θ in the µXRD pattern of the day 15 samples and the absence of other main signals belonging to crystalline mackinawite is best attributed to the presence of a disordered mackinawite-like phase (FeS_am_)^32^. A sharper signal obtained after 6 months at the same position and the presence of other mackinawite signals indicated maturation to crystalline mackinawite. In SEM images, maturation of FeS_am_ aggregates into a more crystalline, curved and wrinkled sheet-like structures was observed after 6 months (Fig. 1f). Bourdoiseau et al.^33^ demonstrated that mackinawite develops a more crystalline structure under ferruginous conditions. The positive influence of Fe^2+^_(aq)_ on mackinawite crystallinity has also been observed in cultures of sulfate-reducing bacteria^15^. Hence, prevailing ferruginous conditions in our Fe/S: 4:1 experiments caused an increase in the crystallinity of mackinawite. However, greigite and pyrite were not detected in these experiments.

### Fe/S: 1:4 experiments: pyrite formation in sulfidic conditions

**Fig. 2:**
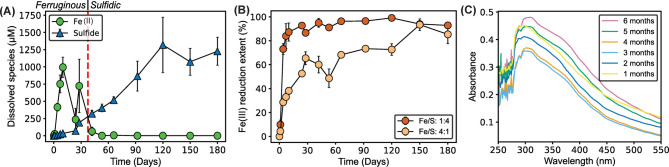
Solution conditions and faster Fe(III) reduction in Fe/S: 1:4 experiments. (**A**) Dissolved Fe(II) and sulfide concentrations in Fe/S: 1:4 experiments. Red dashed line indicates the transition from ferruginous to sulfidic conditions. (**B**) Fe(III) reduction (%) comparing the two different Fe/S ratios experiments. (**C**) UV–Vis spectra for polysulfide detections in Fe/S: 1:4 experiments.

In comparison with the Fe/S: 4:1 experiments, the Fe/S: 1:4 experiments also showed a similar color change from brown to black resulting from ferrihydrite reduction (Supplementary Fig. 1). Dissolved Fe(II) was released faster and accumulated more in these experiments, reaching ~ 1000 µM by day 10 (Fig. 2a). After 40 days, Fe^2+^_(aq)_ decreased to < 65 µM with a concurrent increase in dissolved sulfide, and it remained lower than the detection limit (1 µM) after 2 months. Dissolved sulfide continued to increase until reaching a plateau of ~ 1300 µM on day 120. The crossover on day 40 indicates the ferruginous-sulfidic transition, the transition from Fe^2+^-rich solution to sulfide-rich solution.

The measured total Fe(II)/Fe(total) ratios (dissolved + suspension) indicated faster Fe(III) reduction in the Fe/S: 1:4 experiments (Fig. 2b). Fe(III) reduction rates were calculated by using zero-order kinetics for the first 4 days of Fe/S: 1:4 experiments and 28 days of Fe/S: 4:1 experiments. In Fe/S: 1:4 experiments, Fe(III) reduction was ~ 8 times faster (5.48 mM/day vs. 0.70 mM/day), consistent with faster acetate consumption in the Fe/S: 1:4 experiments (Supplementary Fig. 2). Liu et al.^34^ demonstrated that in the presence of ferrihydrite and S^0^, only one fourth of Fe(III) was reduced by direct attachment of *G. sulfurreducens*, and the rest of the Fe(III) reduction was mediated by an abiotic reaction with sulfide in addition to mackinawite-conducted bioreduction of ferrihydrite. Therefore, faster Fe(III) reduction in experiments with higher sulfur can be explained by the contribution of biogenic sulfide-mediated Fe(III) reduction. While biological ferrihydrite reduction is thermodynamically more favorable than S^0^ under the initial experimental conditions^30^, S^0^ reduction may occur concurrently depending on the spatial distribution of cells and minerals in the system^17^. Furthermore, as Fe^2+^ accumulated in the system, the Gibbs free energy available from Fe(III) reduction becomes lower, favoring the likelihood of S^0^ reduction. When dissolved sulfide is negligible, thermodynamical calculations showed that S^0^ reduction becomes more favorable than ferrihydrite reduction when Fe^2+^_(aq)_ is higher than 55 µM (Supplementary Section 1, Supplementary Fig. 3).

The formation of polysulfide species was followed by UV–Vis spectroscopy, as it is an important intermediate species for pyrite formation. Absorption peaks at 280 and 314 nm indicated the presence of polysulfides in the Fe/S: 1:4 experiments at every sampling point (Fig. 2c)^10^, in contrast to the 4:1 experiments (Fig. 1b). However, no trend (increase or decrease of polysulfide concentration) was observed over time in the experiment. Absolute absorbance values were lower than pyrite formation studies via polysulfide pathway at higher temperatures (> 60 °C)^10,35^, because polysulfide formation kinetics are slower at ambient temperatures^36^. Sulfidic conditions in Fe/S: 1:4 experiments favored the availability of sulfide to interact with S^0^ to form polysulfides^24^, consistent with polysulfide detections in sulfidic zones of meromictic lakes and sediments^26,37–39^.

**Fig. 3:**
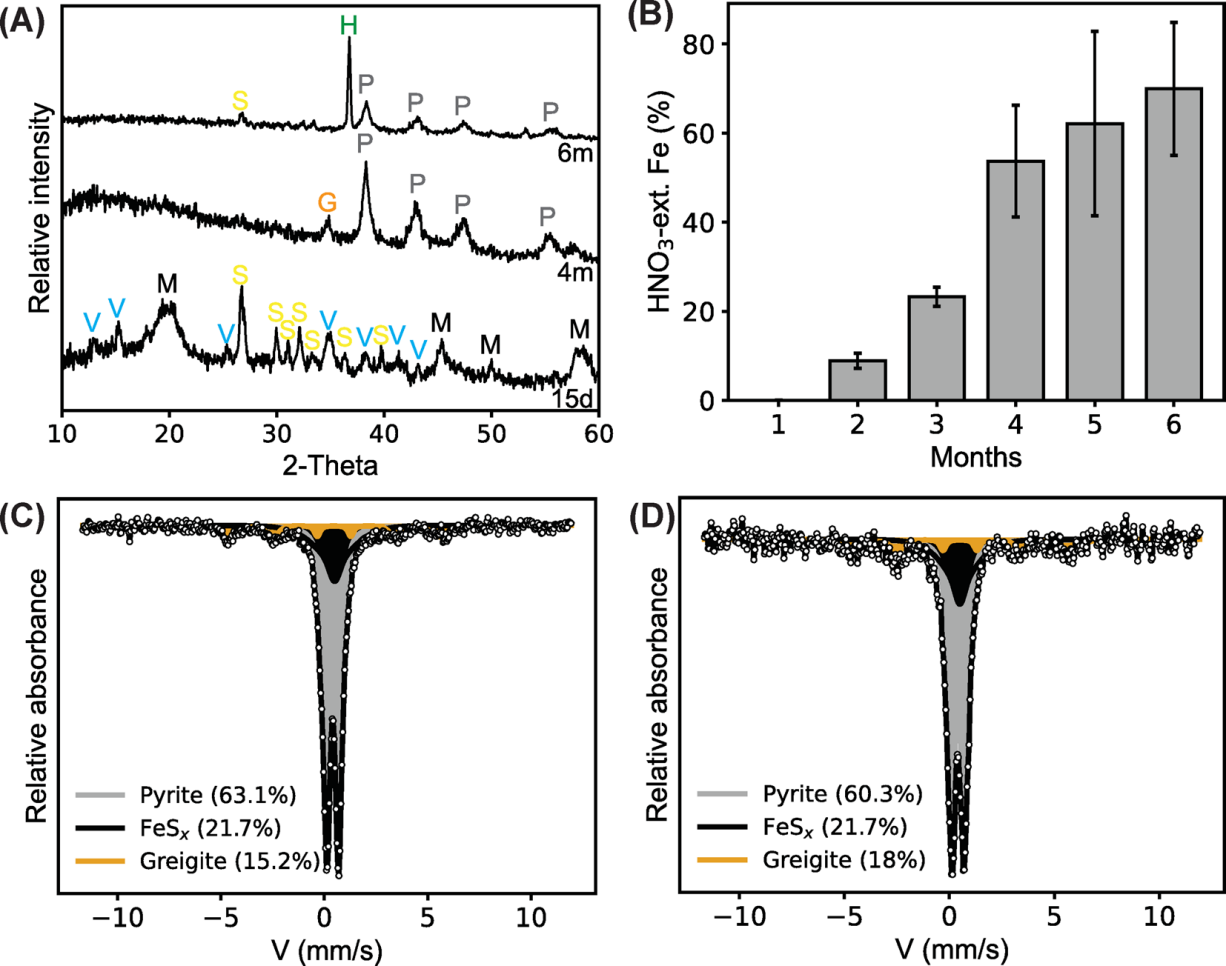
Mineralogy and pyritization extent in Fe/S: 1:4 experiments. (**A**) Mineralogy determined by µXRD analysis. (**B**) HNO_3_-extractable Fe percentage during sequential Fe extraction, representing the pyritization degree. (**C**) Fe mineralogy at the end of the experiment determined by ^57^Fe Mössbauer spectroscopy at 77 K and (**D**) 5 K. d: days, m: months, V: vivianite, M: mackinawite, S: sulfur, G: greigite, P: pyrite, H: halite.

Mineralogical analysis via µXRD revealed the initial precipitation of vivianite (Fe_3_(PO_4_)_2_·8H_2_O) and mackinawite (Fig. 3a). Mackinawite is the first product of the reaction between ferrous iron and sulfide, and it had been observed in numerous studies that investigated the sulfidation of Fe(III) (oxyhydr)oxides biotically^17,40,41^, and abiotically^10,20,35,42^. Vivianite formed by the reaction between the released ferrous iron and the phosphate available in the medium^17^. Vivianite formed only in the Fe/S: 1:4 experiments and not in the 4:1 experiments, even though both experiments were supersaturated with respect to vivianite (Supplementary Section 2, Supplementary Fig. 4). This difference could be explained by differential kinetics for vivianite versus mackinawite formation, as well as in sulfide-mediated vivianite dissolution. While this finding could be important for the fate of phosphorus in the environment, this topic is not the main focus of our study.

Mackinawite and vivianite disappeared subsequently, and greigite and pyrite were detected in the later stages of the experiment (Fig. 3a). Sulfur was still detectable after 6 months via µXRD analysis. Pyrite and greigite formation were confirmed by ^57^Fe Mössbauer spectroscopy at the end of the experiment (Fig. 3c–d; Supplementary Table 1). Pyrite formation and the disappearance of vivianite were related to the solution conditions that drove mineral transformations. Vivianite is not stable under sulfidic conditions, and the transition of ferruginous conditions to sulfidic conditions increases the availability of phosphate by the dissolution of vivianite^15,43,44^. Phosphate release was observed in Fe/S: 1:4 experiments after the ferruginous-sulfidic condition transition (Supplementary Fig. 4a). Sulfidic conditions also favored the formation of polysulfide species, and the presence of polysulfide species suggested pyrite formation via polysulfide pathway with the formation of greigite as an intermediate mineral^23^. HNO_3_-extractable phase (Fe(HNO_3_)/(Fe(HCl) + Fe(HNO_3_)) in sequential Fe extraction was used to track and quantify pyritization. Pyrite formation was detected starting in the second month, representing 8.8% of Fe phases (Fig. 3b). More pyrite continued to form, comprising 69.9% of total Fe in the system after 6 months. The pyrite abundance after 6 months was also determined with ^57^Fe Mössbauer spectroscopy as 63.1 and 60.3% when measured at 77 K and 5 K (Fig. 3c–d), respectively, consistent with the sequential extraction data. The pyrite formation rate in our experiments (0.116 mM/day over 6 months) is in the range of pyritization rates observed in marine sediments^45,46^.

Previous incubations of *G. sulfurreducens* with ferrihydrite and S^0^ together at an Fe/S ratio of 1:1 resulted in the formation of mackinawite^34^, and at a 1:2 ratio resulted in the formation of mackinawite and greigite^17^, but pyrite formation was not observed in both studies. In comparison to the experiments in our study, our results indicate that elemental sulfur abundance is a significant factor affecting biogenic pyrite formation.

**Fig. 4:**
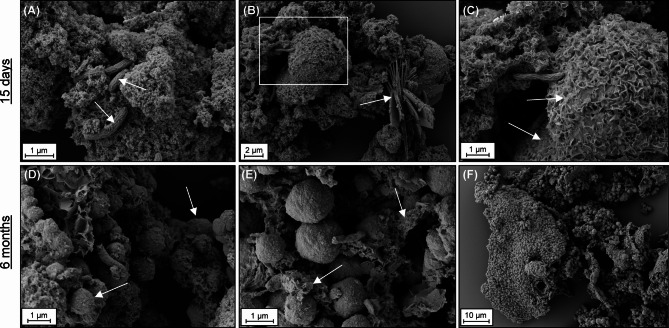
SEM micrographs of the minerals and cells in Fe/S: 1:4 experiments. Micrographs belong to chemically fixed samples on day 15 (**A**–**C**) and after 6 months (**D**–**F**). C is a close up of the area indicated by the white square in B. Arrows in the images represent: Cells in A, vivianite in B, mackinawite encrusted sulfur particles in C, pyrite spherules in D, and the other iron sulfides present with pyrite spherules in E. Individual spherical pyrite particles are present as clusters in F.

Aggregates of mackinawite were observed by SEM within the first 15 days in Fe/S: 1:4 experiments, similar to in Fe/S: 4:1 experiments (Fig. 4a). These initially formed mackinawite minerals were present as distinct aggregates as well as crusts on bigger sulfur particles (Fig. 4b,c). No cell encrustation was observed, irrespective of the experiments or sampling points. Vivianite particles with radial blade-like morphology were detected only at day 15, consistent with µXRD analysis (Figs. 3a and 4b). After 6 months, spherulitic particles ranging from 300 nm to 3 µm were the dominant morphotype in the Fe/S: 1:4 experiments (Fig. 4d–f). We interpreted these particles as pyrite, given their abundance based on µXRD and Mössbauer data, Fe/S ratios (0.46–0.48) of the particles measured by energy dispersive X-ray spectroscopy (EDS) (Supplementary Fig. 5), and the detection of pyrite spherules in other microbial cultures^14,15,47^. Flaky sheet-like structures were also detected in close association with pyrite particles (Fig. 4e). We interpreted these features to be sheets of mackinawite (perhaps with greigite intermixed) based on the morphological similarity to aged mackinawite in our Fe/S: 4:1 experiments (Fig. 1f). Occasionally, clusters composed of individual pyrite spherules were observed, adopting a shell-like structure, reminiscent of mackinawite encrustation of sulfur particles (Fig. 4f).

**Fig. 5:**
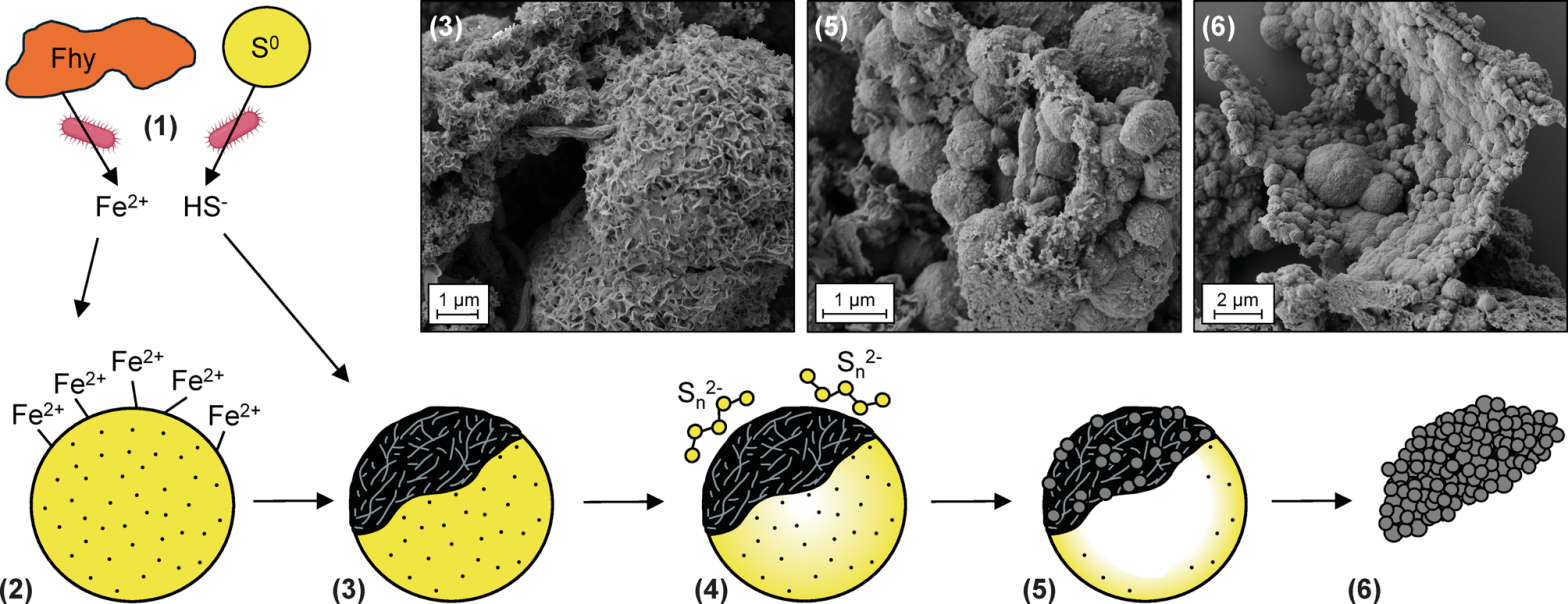
Proposed steps of biogenic pyrite formation through replacement of initial S^0^ particles. (1) Concurrent microbial reduction of ferrihydrite and S^0^. (2) Fe^2+^ adsorption onto sulfur particles. (3) Mackinawite formation on S^0^ particles. (4) Polysulfide enrichment on sulfur microenvironments, and an inside-out dissolution mechanism facilitated by mackinawite’s conductivity. (5) Iron sulfide transformation to pyrite with the consumption of S^0^ and (6) remaining pyrite particles preserving initial sulfur particle shape. SEM micrographs belong to chemically fixed samples.

Based on these observations, we hypothesize a transformation sequence starting from mackinawite encrustation on sulfur particles and subsequent transformation to pyrite clusters while preserving traces of the shape of initial sulfur particles (Fig. 5). For the first part of the sequence, two mechanisms can be proposed for mackinawite encrustation on sulfur particles. Mackinawite could have formed in solution and be deposited on sulfur particles after precipitation, or it could have formed directly on the sulfur particles. We prefer the latter explanation given (1) the tight association between mackinawite and sulfur even after ethanol-based chemical fixation in SEM preparation, (2) negative surface charge of the sulfur particles in experimental solution (− 24.03 ± 2.42 mV), and (3) experiments demonstrating that positively charged Fe^2+^ is adsorbed onto negatively charged S^0^, which is a prerequisite for encrustation (Supplementary Section 3, Supplementary Fig. 6). Due to its conductive properties, the mackinawite coating may accelerate electron transfer between sulfur particle, microbes, and the solution phase, resulting in a reaction front at the surfaces of S^0^ particles^48^. This could favor a dissolution of sulfur inside the sulfur particle via electron transfer, resulting in hollow S^0^ shells. This microenvironment also facilitates polysulfide formation by the nucleophilic reaction between dissolved sulfide and sulfur particles, resulting in polysulfide enrichment around sulfur particles rather than bulk solution^24,49^. This microenvironment is highly favorable for the formation of pyrite grains that adopt the morphology of the initial mackinawite-encrusted S^0^ particles. It must be noted that S^0^ particles were not directly replaced by pyrite grains, given the discrepancy in their sizes (pyrite: 300 nm to 3 µm; S^0^ > 5 µm) (Fig. 4b–e). Rather, pyrite replaced the mackinawite/greigite grains that were arranged in the form of shells around the initial S^0^ particles. Pyrite formation with spherulitic morphology is generally favored at high supersaturation^50^ when nucleation dominates over growth^49^, which can be influenced by the presence of organics^10,15^. Sawlowicz^51^ suggested that the initial pyrite spherules can continuously grow to framboids, and finally to euhedral crystals; but no euhedral or framboidal morphology was observed here.

The pyrite precipitation rate in this study (0.116 mM/day) is in the range of rates determined in previous pyrite formation experiments (0.012–0.367 mM/day)^13,14,46,47,52^. Biogenic pyrite minerals in these studies have a similar spherical/subhedral morphology, presumably as a result of similar kinetics. It is thought that the spherical morphology cannot be used as a biosignature due to recent reports for spherulitic pyrite formation in abiotic setups^49,53^. Instead, in our study we observed that pyrite clusters preserved the morphology of sulfur particles. Hence, pyrite clusters may be considered as a strong signature for pyrite formation within sulfur microenvironments.

### Environmental implications

Sulfate-reducing bacteria (SRB) are generally considered for biogenic pyrite formation, because sulfate is the second most abundant oxidant available for biological activity (28 mM) in seawater^3,29^. Therefore, the abundance of sulfate is related to the significant contribution of SRB on sulfide production at ambient temperatures. However, most of the sulfide (80–90%) is reoxidized by sulfide-oxidizing bacteria or chemical oxidants such as nitrate, manganese(IV) oxides, and Fe(III) (oxyhydr)oxides; and only 10–20% of the sulfide is buried as Fe sulfide minerals or by organic matter sulfurization^54–56^. The abiotic reoxidation of sulfide mostly produces elemental sulfur rather than sulfate^26,57^. Additionally, some microorganisms are capable of reducing sulfate not to sulfide, but to elemental sulfur (e.g., Sulfammox bacteria)^58^. Therefore, S^0^ is a potential key (secondary) source of sulfide in sediments. In case of sulfide production by SRB, sulfide needs to diffuse to reach S^0^ particles to form polysulfides in sedimentary environments. However, polysulfide consumption^59^ is expected to be faster than sulfide diffusion^60^. On the other hand, sulfur-reducing bacteria are located close to sulfur particles, which may favor the accumulation of polysulfides. The difference between these two processes controlling polysulfide formation and consumption may indicate the role of microhabitats belonging to sulfur-reducing bacteria on biogenic pyrite formation. Our results show that sulfur-reducing bacteria could replace the role of SRB as (poly)sulfide suppliers for pyrite formation, and elemental sulfur serves a dual function as polysulfide hotspots and as templates for the formation of pyrite clusters. In natural environments, the heterogenous distribution of elemental sulfur would favor the formation of microenvironments. Wang and Morse^50^ previously observed heterogeneous distribution of pyrite particles in their experiment. They proposed that elemental sulfur distribution and related polysulfide formation are the factors controlling pyrite formation due to higher pyrite formation kinetics with the addition of elemental sulfur. Pyrite formation models in Black Sea sediments suggests pyrite formation via polysulfide pathway as long as elemental sulfur is present in the system^61^. Similarly, high sedimentation rates also create microenvironments rich in Fe(III) (oxyhydr)oxide minerals as an oxidant for sulfide, resulting in the formation of pyrite via elemental sulfur as an intermediate^62^. Additionally, sulfide-oxidizing and Fe(II)-oxidizing microorganisms can form microbial mats consisting of elemental sulfur and Fe(III) (oxyhydr)oxide minerals^63^. Sulfur in microbial mats could be a hotspot for S^0^ formation, which then could be another hotspot for pyrite formation after the burial of the mat. In this study, mackinawite encrustation and subsequent pyritization on sulfur particles confirmed the role of sulfur microenvironments as potential hotspots for pyrite formation.

While sulfate is a common electron acceptor for life on today’s Earth, it was not available at high concentrations in Archean oceans. Models suggest that the abundance of sulfate in Archean seawater was less than 2.5 µM^64^. The ∆^33^S values of Archean pyrite in 3.2 Ga sediments in Barberton Greenstone Belt, Canada indicate elemental sulfur as the primary sulfur source^65^. Additionally, ∆^33^S anomalies in sulfide minerals in marine sulfate deposits from Dresser Formation, Australia (3.4 Ga) demonstrates the preference of Archean microorganisms for elemental sulfur rather than sulfate^66^. The biogenic formation mechanism in our experiments may be relevant for sulfate-poor environments such as the Archean Earth. Investigating the morphology and isotopic compositions of pyrite formed on elemental sulfur particles could have implications to understanding both modern and ancient environments, but the preservation of such signatures through geological timescales needs further investigation.

## Conclusions

In this study, we reported pyrite formation by the Fe(III)- and S^0^-reducing bacterium *G. sulfurreducens* in the presence of both ferrihydrite and S^0^. Pyrite formation was controlled by the Fe/S ratio, with none forming at Fe/S: 4:1 and a significant amount forming at Fe/S: 1:4, achieving ~ 70% yield at a formation rate of 0.116 mM/day, comparable to rates in marine sediments. Mackinawite was formed first as coatings around elemental sulfur, before transforming to spherulitic pyrite clusters over time. We propose that elemental sulfur acts as hotspots for pyrite formation by providing microenvironments rich in polysulfides and surface templates for precipitation. This provides an explanation for spatially heterogenous pyrite formation in nature, as controlled by S^0^ availability, with relevance both to modern and ancient environments.

## Methods

### Mineral synthesis and microbial cultivation

The ferric oxyhydroxide mineral ferrihydrite and elemental sulfur were used in experiments as Fe and S sources. 2-line ferrihydrite was synthesized by dissolving 40 g Fe(NO_3_)_3_·9H_2_O in 500 mL Milli-Q water^67^. The pH of the solution was adjusted to 7.3 via titration with 1 M KOH. The resulting suspension was incubated at room temperature for 2 h, and the pH was readjusted to 7.5. Then, minerals were washed four times with Milli-Q water by centrifugation for 10 min at 5000 g. After the last washing step, ferrihydrite was resuspended with 200 mL Milli-Q water and flushed with N_2_/CO_2_ (80:20) mix. All the steps were performed under sterile conditions. The final ferrihydrite concentration was determined by the Ferrozine assay following dissolution in 1 M HCl. Commercially available elemental sulfur (Sigma-Aldrich, product number 13803) was used in the experiments. Prior to experiments, sulfur was firstly flushed with N_2_ (100%) gas for O_2_ removal and sterilized by tyndallization for 45 min at 80 °C for three consecutive days.

*Geobacter sulfurreducens* PCA (DSM 12127) is an obligate anaerobe, Fe(III)- and S^0^-reducing bacterium that was first isolated from a ditch surface sediment^68^. For bacterial cultivation, the base medium containing 4.4 mM KH_2_PO_4_, 5.6 mM NH_4_Cl, 2 mM MgSO_4_.7H_2_O, 0.7 mM CaCl_2_·2H_2_O, SL10 trace elements solution (1 mL/L), 7 vitamin solution (1 mL/L), and selenite tungstate solution (1 mL/L) was prepared and sterilized by autoclaving at 121 °C for 15 min. The solution was immediately flushed with N_2_/CO_2_ mix (80:20) after autoclave and cooled to room temperature. 30 mM NaHCO_3_ was added as a buffer, and the final pH was adjusted to 7.0 with 1 M HCl. The growth medium was completed by supplying 25 mM acetate and 40 mM fumarate as an electron donor–acceptor couple. Cultures were prepared as 100 mL volume in a 200 mL serum bottle and transferred three times before used in the experiments at 28 °C for 5 days.

### Experimental setup

The preparation and sampling of the experiments were performed under sterile and anoxic conditions. Batch experiments were performed in 50 mL volume dispensed into 100 mL serum bottles under N_2_/CO_2_ atmosphere. The base medium prepared for bacterial cultivation was also used as the experimental solution. Two different Fe/S ratios (4:1 and 1:4) were tested for pyrite formation. Ferrihydrite concentration was kept constant at 30 mM while elemental sulfur was supplied at either 7.5 mM (12 mg) or 120 mM (192 mg). Ferrihydrite and elemental sulfur were used as electron acceptors while 25 mM Na-acetate was supplied as an electron donor. A volume of 5 mL *G. sulfurreducens* culture (OD_600_ = 0.7) was added to biotic experiments. The serum bottles were capped with rubber stoppers and sealed with aluminum crimps. Three biotic replicates and two abiotic control replicates were prepared, and all the bottles were incubated at 28 °C for 6 months.

### Geochemical analyses

Experiments were sampled under anoxic conditions in the glovebox. 2 mL aliquots were centrifuged first at 12,100 g for 1 min to separate mineral and solution phases. Solution chemistry was followed by measurements of dissolved Fe, dissolved sulfide, and acetate concentration. Samples used for dissolved Fe and acetate concentrations were acidified by dilution with 1 M HCl to prevent Fe(II) oxidation. Fe^2+^_(aq)_ and Fe(tot)_(aq)_ (total Fe_(aq)_ = Fe^2+^_(aq)_ + Fe^3+^_(aq)_) were determined by Ferrozine assay^69^. Acetate concentration was measured by high-performance liquid chromatography (HPLC) analysis in a Shimadzu Prominence HPLC, which was equipped with an LC-20AT solvent delivery unit, a CTO-10ASvp column oven, and a RID-20a refractive index detector. The remaining unacidified supernatant was used for dissolved sulfide and polysulfide measurements. Dissolved sulfide concentration was determined by methylene blue method^70^ after fixation of the sulfide with 1 M Zn-acetate solution (1:1). Polysulfide presence was followed by ultraviolet–visible (UV–Vis) spectroscopy with an absorbance spectrum for the range between 250 and 550 nm wavelength^31^. The resulting spectra were normalized by using the spectra of the base medium which does not contain any Fe or S.

Mineral phase obtained after centrifugation was used to track Fe(III) reduction and pyritization via sequential Fe extraction method. First, 1 M HCl was added to the particles to dissolve HCl-extractable Fe minerals in the system such as ferrihydrite, mackinawite, greigite, and vivianite. After incubation overnight at room temperature, Fe(II) and Fe(tot) concentrations in the extract were measured by Ferrozine analysis to determine Fe(III) reduction. In this case, the sulfide fraction present in solution or released from the iron sulfide minerals can reduce additional Fe(III) during HCl extraction, resulting in elevated Fe(II) concentrations^20^. Thus, the measured Fe(II)/Fe(tot) ratios could be an overestimation but nonetheless tracked the overall reduction of the system. The remaining particles were treated with 8 M HNO_3_ to dissolve HCl-insoluble pyrite. Fe concentration of the HNO_3_-extractable phase was used to follow pyritization through time. Different amounts of particles were sampled during analysis, as evidenced by Fe(tot) that varied from 26 to 37 mM even within a single bottle (data not shown). Thus, Fe concentration of the HNO_3_-extractable phase at each time point was normalized to the initial Fe(tot) concentration (30 mM) to track the absolute amount of pyritization.

### Mineralogical analyses

µXRD measurements and Mössbauer analysis were used for the identification of the mineralogy in the experiments. Color changes resulting from mineral transformation were followed by daily observation, and magnetism in the solid phase was checked with a hand magnet to check for the presence of magnetic minerals.

Aliquots of 2 mL were sampled for µXRD analysis and washed three times with anoxic Milli-Q water by centrifugation at 12,100 g for 1 min. Minerals were dried for 1–3 days in the glovebox and stored in anoxic condition. µXRD raw data were collected with a Bruker’s D8 Discover GADDS XRD2 micro-diffractometer with a standard sealed tube and Co-anode (Co Kα radiation with 0.179 nm wavelength) at 30 kV/30 mA. Measurements were performed for 240 s at two detector positions, 15° and 40°. X-ray diffractograms were analyzed with Match! Software (Crystal Impact, Bonn, version 3.11.5.203) with the Crystallography Open Database (COD-Inorg REV211633 2018.19.25).

For ^57^Fe Mössbauer spectroscopy, 10 mL aliquots were filtered through 0.22 µm pore size mixed cellulose ester filters and wrapped with O_2_-impermeable Kapton® tapes. Samples were prepared in the glovebox under anoxic conditions and then stored at − 20 °C. For analysis, samples were inserted into a closed-cycle exchange gas cryostat (Janis Research, USA) under He backflow to minimize air exposure. Mössbauer spectra were collected at 77 K and 5 K using a constant acceleration drive system (WissEL, Germany) in transmission mode with a ^57^Co/Rh source. Raw data were calibrated against a 7 µm thick ^57^Fe foil that was measured at room temperature. Further data analysis was performed with Voigt Based Fitting (VBF) routine^71^ by using Recoil software (University of Ottawa). The half width at half maximum (HWHM) was constrained to 0.138 mm s^−1^ during fitting.

The surface charge of S^0^ was determined via the Zetasizer Nano using 120 mM S^0^ in base growth medium as a sample. The number of runs, attenuation and voltage were determined automatically by the instrument. The measured electrophoretic mobility was converted to surface charge (Zeta potential) using the Smoluchowski method. Four repeat measurements were performed and averaged.

### Morphological analyses

Scanning electron microscopy was used to investigate mineral morphology and cell-mineral interactions. Elemental information was collected for the samples from Fe/S: 4:1 and 1:4 experiments on day 15 and at 6 months via an Oxford Instrument energy dispersive spectrometry detector attached to the SEM. Two different methods were applied for sample preparation: (1) to maintain anoxic conditions of minerals and (2) to preserve cell structures. In the first method, SEM samples were washed three times with Milli-Q water via centrifugation at 12,100 g for a minute, and dried on aluminum sample holders with adhesive carbon tabs. After removing samples from the glovebox, they were immediately coated with 8 nm of Pt by using a BAL-TEC™ SCD 005 sputter coater. Those samples were used for EDS analysis to obtain elemental information of the particles. In the second method, samples were fixed in 2.5% electron microscopy-grade glutaraldehyde in original sample medium at 4 °C overnight in Eppendorf tubes. After the incubation samples were washed three times with Milli-Q water via similar centrifugation process as in the first method. 50 µL of each sample were added onto poly-L-lysine coated cover glass slides, positioned in a 24-well plate, and incubated for 30 min. Next, 500 µL of 25% ethanol was added to the samples and left for 15 min. After removing 25% ethanol, the sequential ethanol dehydration process was repeated once for 50% and 75% by 15 min of incubations and three times for 100% ethanol by 30 min of incubations. After that, the samples were incubated in 500 µL hexamethyldisilazane (HMDS)/ethanol mix (50:50) for another 30 min, then replaced by 500 µL HMDS and left for 30 min again. In a final step, 250 µL of fresh HMDS were added to the wells and the samples were left to air dry in a fume hood overnight (with the well plate cover lid slightly opened) and coated with Pt on the next day as described in the first method. SEM images and EDS spectra were obtained by using a Zeiss Crossbeam 550L Scanning Electron Microscope equipped with an Oxford Instrument Energy Dispersive Spectrometer (UltimMax 100, Oxford Instrument, Abingdon, United Kingdom). Micrographs for the surface investigation of the samples were collected with an acceleration voltage of 2 kV and a probe current of 100 pA at a working distance of 5 mm using the SeSi detector. EDS point scans were performed at a working distance of 5 mm using an acceleration voltage of 15 kV and a probe current of 2.1–2.3 nA, with a detector deadtime value of around 35%.

## Electronic supplementary material

Below is the link to the electronic supplementary material.

Supplementary Material 1

## Data Availability

The data produced and used in this study are available within the paper and its Supplementary Materials. Any additional information can be provided by corresponding authors upon request.
