# Supplementary material for: Sulfur microenvironments as hotspots for biogenic pyrite formation
Source: Sci Rep. 2025 Jun 20;15:20148. doi: 10.1038/s41598-025-05178-8 (PMC12181240; doi:10.1038/s41598-025-05178-8)
Supplement: Supplementary file 1 — Supplementary Material 1 [file 41598_2025_5178_MOESM1_ESM.docx]

# Supplementary Information

# Sulfur Microenvironments as Hotspots for Biogenic Pyrite Formation

Fatih Sekerci^1^, Stefan Fischer^2^, Prachi Joshi^1^, Stefan Peiffer^3^, Andreas Kappler^1,4^,
Muammar Mansor^1*^

^1^Geomicrobiology, Department of Geosciences, University of Tuebingen, Germany

^2^Tuebingen Structural Microscopy Core Facility, University of Tuebingen, Germany

^3^Hydrology, Bayreuth Center for Ecology and Environmental Science, University of Bayreuth, Germany

^4^Cluster of Excellence: EXC 2124: Controlling Microbes to Fight Infection, Tuebingen, Germany

*Corresponding Author: muammar.mansor@uni-tuebingen.de


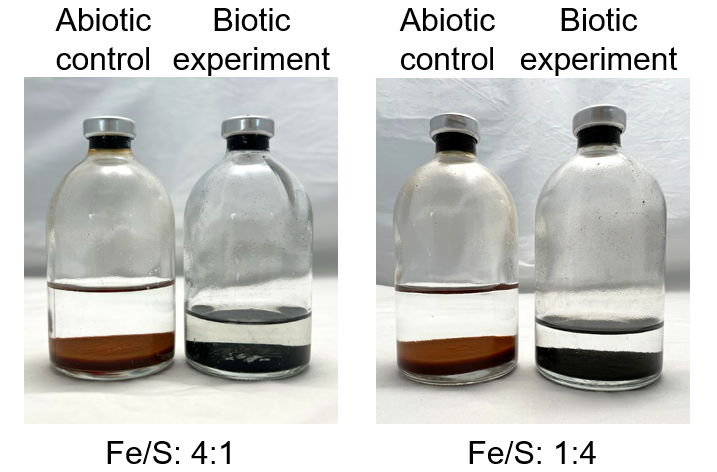


**Supplementary Fig. 1 Color change in Fe/S:4:1 (left) and Fe/S: 1:4 experiments (right) after 15 days.** Ferrihydrite (brown) stayed stable in abiotic control experiments which contain only ferrihydrite and elemental sulfur with medium. In biotic experiments, ferrihydrite was reduced by *G. sulfurreducens* and mackinawite (black) formation was observed by color change.


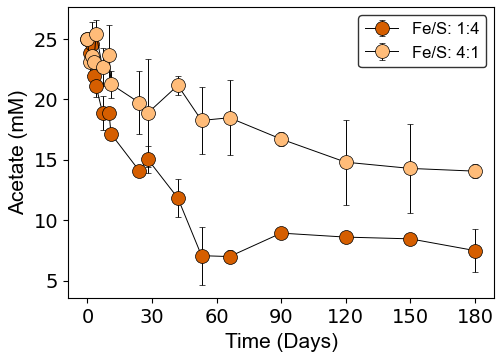


**Supplementary Fig. 2 Acetate concentrations in the experiments.** Acetate was consumed faster in Fe/S: 1:4 experiments, consistent with the higher concentration of electron acceptors (ferrihydrite + 120 mM S^0^). The trend is also consistent with faster total Fe(III) reduction (dissimilatory bacterial Fe(III) reduction + sulfide-mediated abiotic Fe(III) reduction) in these experiments (Fig. 2b).

**Supplementary Section 1. Available energy from ferrihydrite and sulfur reduction**

Acetate-coupled ferrihydrite reduction (Reaction S-1) and sulfur reduction (Reaction S-2) were used to calculate the available energy for bacterial metabolism throughout the experimental period.

8Fe(OH)_3_ + CH_3_COO^-^ + 15H^+^ → 8Fe^2+^ + HCO_3_^-^ + 20H_2_O ∆G° = -573.4 kJ/mol (S-1)

4S^0^ + CH_3_COO^-^ + 5H_2_O → 4HS^-^ + 2HCO_3_^-^ + 5H^+^ ∆G° = 192.3 kJ/mol (S-2)

Dissolved ferrous iron, sulfide, and acetate concentrations were included in the Nernst equation (Equation S-3), where ∆G_rxn_ is the Gibbs free energy of the reaction, ∆G° is the standard Gibbs free energy, R is the universal gas constant 8.314 J/mol*K, T is temperature, and Q is the reaction quotient. pH is assumed to be 7 throughout.

∆G_rxn_ = ∆G° + RTlnQ (S-3)

Standard Gibbs free energies for the reaction were obtained from Flynn et al.^1^. Speciation of the reactants and products were calculated by using PHREEQC software^2^ with the phreeqc.dat database.

Ferrihydrite reduction provides more available energy than sulfur reduction coupled with acetate oxidation under standard conditions^1^. However, under our experimental conditions, sulfur reduction became more favorable once Fe^2+^_(aq)_ accumulated within the first 4 days of both experiments (Supplementary Fig. 3a-b). Sulfur reduction generally became more favorable than ferrihydrite reduction in the presence of 55 µM Fe^2+^_(aq)_, assuming negligible dissolved sulfide (Supplementary Fig. 3c).With the accumulation of dissolved sulfide, ferrihydrite reduction became more favorable again after 120 days for Fe/S: 4:1 experiments and 24 days for Fe/S: 1:4 experiments. However, at those time points, no Fe(III) was available for reduction due to its complete reduction in the earlier stages (Fig. 2b).


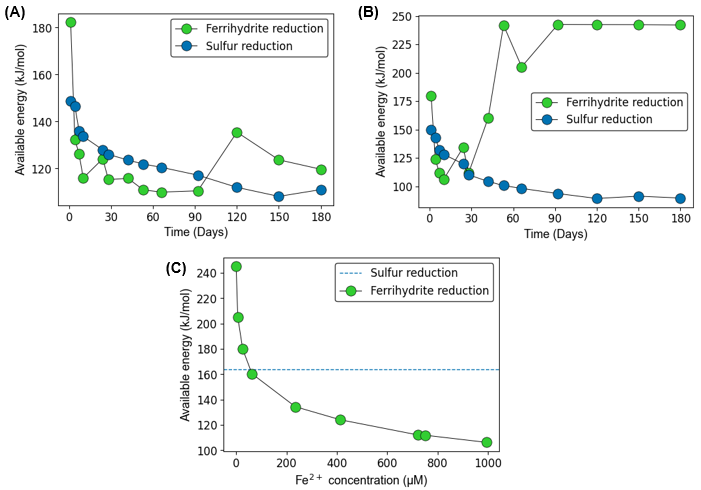


**Supplementary Fig. 3. Available energy for microbial ferrihydrite and sulfur reduction coupled to acetate oxidation.** (A) Available energy related to solution chemistry for ferrihydrite and sulfur reduction for *Geobacter sulfurreducens* through the experimental period starting at day 1. (A) Fe/S: 4:1, and (B) Fe/S: 1:4 experiments. (C) A theoretical illustration of the crossover point at which sulfur reduction becomes more favorable than ferrihydrite reduction with increasing dissolved ferrous iron. The crossover point is ~55 µM Fe^2+^_(aq)_ in our experiment.

**Supplementary Section 2. Phosphate concentration and solution saturation states for mackinawite and vivianite**

Dissolved phosphate concentration in the experiments were determined via the phosphomolybdate colorimetric assay^3^ (Supplementary Fig. 4a). While most of the phosphate was associated with the solid phase due to phosphate mineral formation (e.g. vivianite)^4^ or adsorption to ferrihydrite^5^ in Fe/S: 4:1 experiments; the ferruginous-sulfidic conditions transition in Fe/S:1:4 experiments was accompanied by a release of phosphate again to solution.

Dissolved phosphate, ferrous iron and sulfide concentrations were used to calculate solution saturation indices (SI) with respect to mackinawite and vivianite (Supplementary Fig. 4b-c). Calculations were performed by using PHREEQC software^2^ with the formula (Reaction S-4):

SI = log(IAP/K_sp_) (S-4)

where IAP is the ion activity product, and K_sp_ is the solubility product of corresponding mineral.

The results indicate that mackinawite and vivianite were always supersaturated in Fe/S: 4:1 experiments, and Fe/S: 1:4 experiments while ferruginous conditions were present. After the consumption of Fe^2+^_(aq)_, solutions were undersaturated.


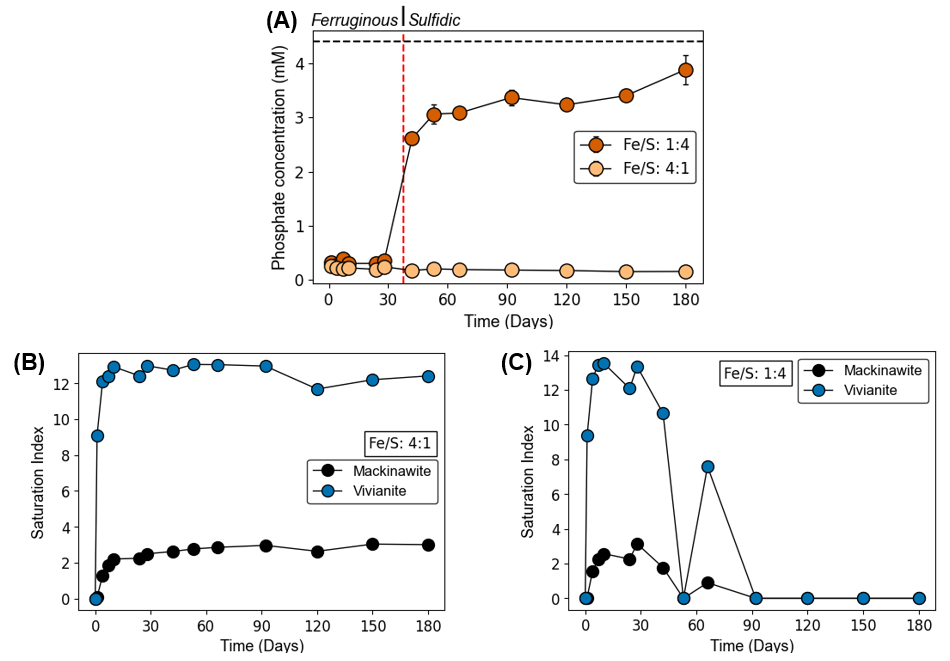
 **Supplementary Fig. 4. Mackinawite and vivianite saturation states in Fe/S: 1:4 and 4:1 experiments.** (A) Dissolved phosphate concentrations. Black dashed line indicates the total phosphate concentration in the culture medium (4.4 mM). Red dashed line indicates the ferruginous-sulfidic conditions transition in Fe/S: 1:4 experiments derived from solution chemistry data (Fig. 2a). Solution saturation index with respect to mackinawite and vivianite in (B) Fe/S: 4:1 and (C) Fe/S: 1:4 experiments.

**Supplementary Table 1.** Parameters used for Mössbauer spectroscopy for the 6^th^ month sample of the Fe/S: 1:4 experiment. CS: center shift, QS/ε: quadrupole splitting, H (T): Hyperfine field, χ^2^: goodness of fit^6,7,8^.

| T (K) | Site | CS (mm/s) | QS/ε (mm/s) | H (T) | Relative area (%) | χ^2^ |
| --- | --- | --- | --- | --- | --- | --- |
| 77 K | Pyrite | 0.40 | 0.61 |  | 63.10 | 0.99 |
|  | Greigite 1 | 0.39 | 0.00 | 24.91 | 3.10 |  |
|  | Greigite 2 | 0.50 | 0.03 | 31.87 | 12.10 |  |
|  | FeS_x_ | 0.50 | 0.00 | 6.32 | 21.70 |  |
| 5 K | Pyrite | 0.40 | 0.63 |  | 60.30 | 0.75 |
|  | Greigite 1 | 0.39 | 0.00 | 29.00 | 5.70 |  |
|  | Greigite 2 | 0.54 | 0.03 | 31.79 | 12.30 |  |
|  | FeS_x_ | 0.50 | 0.00 | 5.58 | 21.80 |  |

**
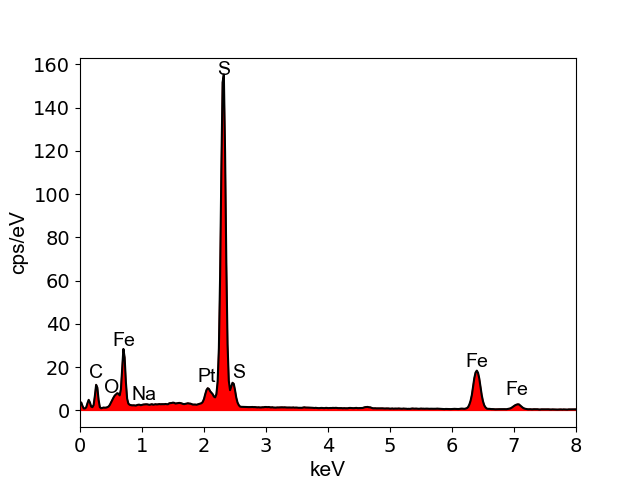

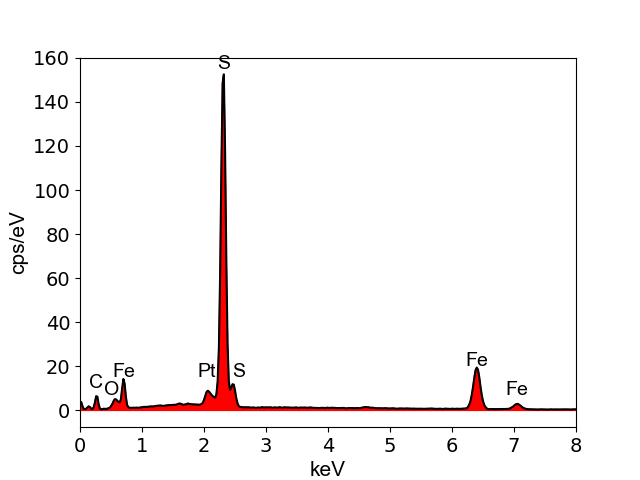

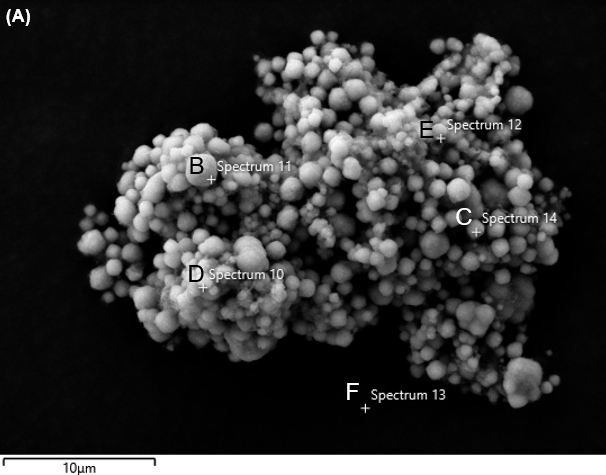
**

**(C)**

**(B)**

**
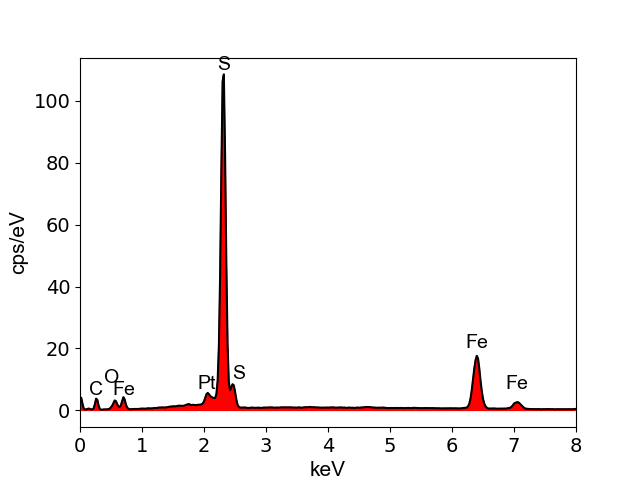
**

**(E)**

**(D)**

**
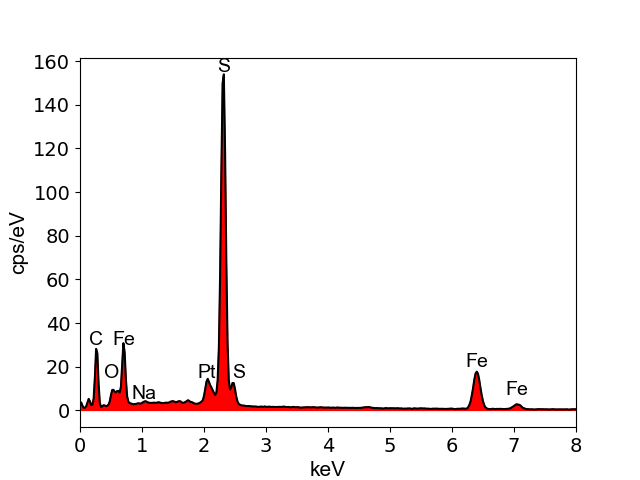
**

**
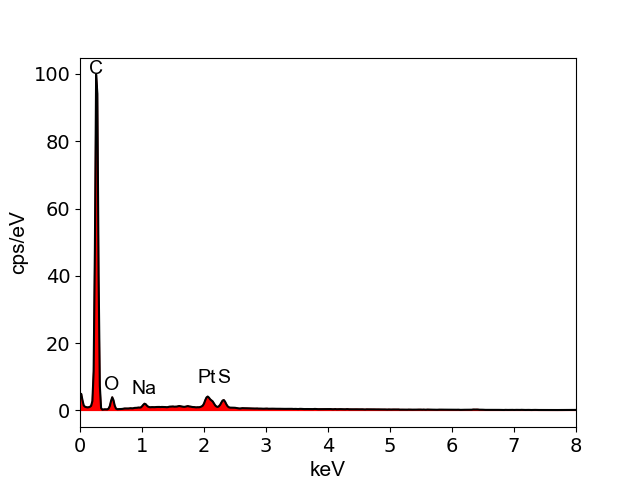
**

**(F)**

**Supplementary Fig. 5. EDS spectra of the particles in biotic Fe/S: 1:4 experiments after 6 months.** (A) SEM image at 15 kV for 6^th^ month samples from Fe/S: 1:4 experiments. (B-D) EDS spectra of the particles consistent with Fe/S molar ratio of pyrite (Fe/S= 0.46-0.48, Fe/S_Pyrite_ = 0.5). (E) EDS spectra of the particles with a Fe/S ratio (0.61) between pyrite and other iron sulfide minerals (Fe/S_Mackinawite_ = 1, Fe/S_Greigite_ = 0.75). (F) EDS spectra for carbon background of the sample holder.

**Supplementary Section 3. Fe^2+^ adsorption onto sulfur particles**

Fe^2+^ adsorption onto sulfur particles was evaluated by the addition of 1 mM Fe^2+^_(aq)_ in the experimental medium or HEPES buffer, and tracking the Fe^2+^_(aq)_ and Fe(II)_solid_ concentrations.

For the experiments with experimental medium (composition described in methods section), FeCl_2_.4H_2_O was dissolved in 50 mL of the medium. After the sampling for t_0_, bottles were incubated overnight to approach equilibrium for Fe(II) mineral (e.g. vivianite and siderite (FeCO_3_)) formation with phosphate and carbonate available in the medium. The next day, the slurry was sampled to quantify total Fe^2+^ (solution + solid phase after the extraction in 1 M HCl) and dissolved Fe^2+^ by Ferrozine assay. Ferrozine assay was applied for both Fe(II) and Fe(total), and no Fe(III) was detected. Mass balance was observed after the first day and it was concluded that nearly half of the Fe^2+^ was consumed for Fe(II) mineral formation (HCl-extractable phase for possible vivianite and siderite formations) (Supplementary Fig. 6a). Then, 192 mg (120 mM) S^0^ was added to the system, and the bottles were incubated overnight. The following day, total HCl-extractable Fe and dissolved Fe were measured again. Interestingly, mass balance between the initial Fe^2+^_(aq)_ and the final Fe^2+^_(aq)_ and Fe(II)_solid_ was not observed. About 22% of Fe precipitated as Fe(II) minerals and another 8% remained in the solution (Supplementary Fig. 6a). The other 70% could not be recovered via HCl, and we interpret this to represent the fraction adsorbed on or associated with sulfur particles. Part of this uncertainty could be due to the difficulty in homogenously sampling large S^0^ particles. The exact association between Fe^2+^ and S^0^ is unknown and warrants further investigation, but it shows that Fe^2+^ could indeed be associated with S^0^, lending support to our encrustation mechanism.

In order to exclude the compounding effect of Fe(II) mineral formation, we investigated the association between 1 mM Fe^2+^ and 120 mM S^0^ in 50 mL HEPES buffer at pH 7. After 24 hours, it was observed that Fe^2+^ remained in the solution, and no significant adsorption onto S^0^ particles occurred (Supplementary Fig. 6b). The contrast between the experimental medium and HEPES buffer may be explained by the presence of organic molecules (e.g. acetate, vitamins) and trace metals in the experimental medium. We hypothesize that organic molecules and/or trace metals facilitate the adsorption of Fe^2+^ to S^0^ in our study via promotive co-sorption effects^9^.


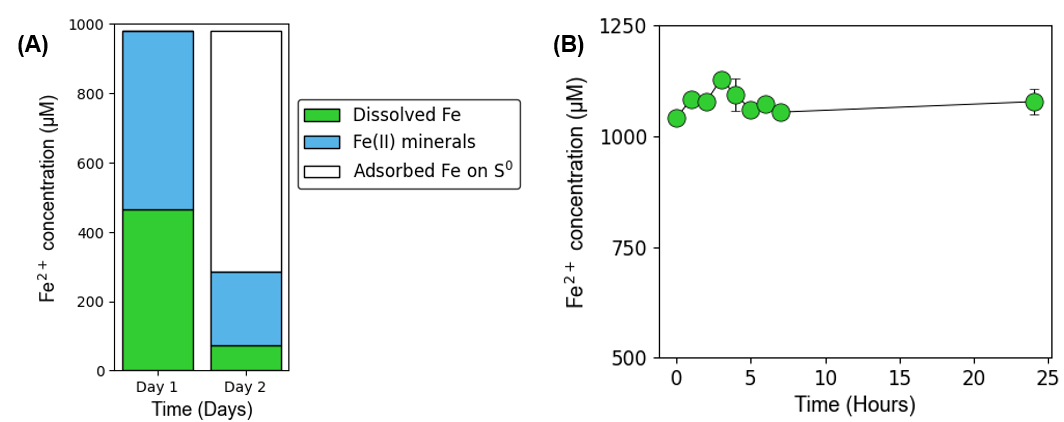


**Supplementary Fig. 6. Fe^2+^ adsorption onto sulfur particles.** (A) Fe phases in Fe^2+^ adsorption experiments with experimental medium. (B) Dissolved Fe^2+^ concentration in the adsorption experiments with HEPES buffer.

## Supplementary References

1. Flynn, T. M., O’Loughlin, E. J., Mishra, B., DiChristina, T. J. & Kemner, K. M. Sulfur-mediated electron shuttling during bacterial iron reduction. *Science* **344**, 1039-1042 (2014).
2. Parkhurst, D. & Appelo, C. *Description of Input and Examples for PHREEQC Version 3 – A Computer Program for Speciation, Batch-reaction, One-dimensional Transport, and Inverse Geochemical Calculation U.S. Geol. Survey Water Resources Investigations Report* (2013).
3. Murphy, J. & Riley, J. P. A modified single solution method for the determination of phosphate in natural waters. *Anal. Chim. Acta* **27**, 31-36 (1962).
4. Bronner, R. et al. Co-reduction of Fe(III) and S0 drives Fe-S biomineral formation and phosphate mobilisation. *Geochem. Persp. Let.* **24**, 27-32 (2023).
5. Antelo, J., Arce, F. & Fiol, S. Arsenate and phosphate adsorption on ferrihydrite nanoparticles. Synergetic interaction with calcium ions. *Chem. Geol.* **410**, 53-62 (2015).
6. Wan, M., Schröder, C. & Peiffer, S. Fe(III):S(-II) concentration ratio controls the pathway and the kinetics of pyrite formation during sulfidation of ferric hydroxides. *Geochim. Cosmochim. Acta* **217**, 334–348 (2017).
7. Thiel, J., Byrne, J. M., Kappler, A., Schink, B. & Pester, M. Pyrite formation from FeS and H _2_ S is mediated through microbial redox activity. *Proc. Natl. Acad. Sci.* **116**, 6897–6902 (2019).
8. Schröder, C. et al. Identification of Mackinawite and Constraints on Its Electronic Configuration Using Mössbauer Spectroscopy. *Minerals* **10**, 1990 (2020).
9. Yan, Y. et al. Co-sorption of metal ions and inorganic anions/organic ligands on environmental minerals: A review. *Sci. Total. Environ.* **803**, 149918 (2022).
